# Soil Organic Matter and Nutrient Levels in Outdoor Runs in Organic Laying Farms

**DOI:** 10.3390/ani13030401

**Published:** 2023-01-25

**Authors:** Michele Zoli, Paolo Mantovi, Paolo Ferrari, Lorenzo Ferrari, Valentina Ferrante

**Affiliations:** 1 Department of Environmental Science and Policy, University of Milan, 20133 Milan, Italy; 2 Fondazione CRPA Studi Ricerche, 42121 Reggio Emilia, Italy

**Keywords:** free-range system, hen runs, nitrogen, phosphorus, organic matter

## Abstract

**Simple Summary:**

Organic egg production is growing more and more, and consumers are increasingly attentive to sustainability issues and animal welfare. In organic production, laying hens must have continuous daytime access to outdoor runs. However, the management of this area is not always easy due to the high load of nutrients due to the feces of the hens. This work, including the FreeBirds project, aims to assess the nutrient load of the free range of three different Italian organic farms in three different contexts. The results show that, in all three farms, there is a high load of nitrates and phosphorus in the areas closest to the hen house, while for other chemical parameters, no clear trend was identified. This study shows that further developments are necessary to make the use of free range more homogeneous to avoid nutrient overloads that could cause high impacts, especially freshwater eutrophication.

**Abstract:**

To evaluate the nutrient load due to the grazing of laying hens in outdoor runs, monitoring of soil characteristics was conducted in three Italian organic farms. For each farm, soil samples were taken from three increasing distances from the hen house and two depths, and different chemical parameters were evaluated. The comparison among the results from the different distances shows that N-NO_3_ and Olsen P are the most affected parameters by hen feces: both present high values with a statistically significant difference in the area close to the poultry house and for the most superficial layer. Even TKN and TOC show significant differences between the concentrations of the first layer (more concentrated) and those of the second layer (less concentrated). In general, the surface soil layer closest to the chicken house is the portion of the outdoor run most affected by chicken droppings and represents the most critical point in terms of potential environmental impact. Therefore, it is necessary to intensify the management of the outdoor run with tools that can facilitate the grazing of animals and with vegetation that can absorb nutrients by limiting leaching and runoff.

## 1. Introduction

Eggs are one of the most consumed animal products and represent an important source of high-quality protein and micronutrients in the human diet [1]. In Europe, 6.7 million tons of eggs are produced annually [2]. In this context, free-range systems are becoming increasingly important because providing animals with constant access to the outdoors can improve their well-being and health. Free-range systems account for about 18% of European egg production, with 67.4 million laying hens in Europe. Of these, about 34% are raised with the organic method [2].

In organic livestock production, improving animal welfare is a key aspect, and this includes spending time outdoors. According to EU regulation requirements [3], free-range laying hen rearing is characterized by continuous daytime access to outdoor runs, with at least 4 m^2^/hen of open space. Moreover, as there is a growing concern for animal welfare among consumers as well, according to some authors [4], free access to outdoor runs maximizes the animals’ behavioral freedom [5]. The latter is a key indicator of their welfare status and a source of information on their perception of their housing conditions [6]. In addition to this, a lower incidence of keel bone fractures in laying hens has been found in animals that frequently use the outdoor range [7]. For all these reasons, this production system is increasingly applied. However, it is necessary to take into account the possible environmental effects that this system may imply, such as the load of soil nutrients (especially nitrogen and phosphorus). In fact, it often happens that animals do not feel safe in outdoor ranges because the latter are not well managed. This leads the laying hens to stay in groups very close to the shed, causing a buildup of nutrients in a limited area [5]. In addition, the concentrated presence of the animals often makes this area bare of vegetation. These conditions differ from a natural ecosystem, in which the rate of nutrient removal by vegetation, vegetation removal by animals, and nutrient return in the form of feces and urine is naturally balanced. In contrast, in organic hen farms, N and P excretion generally exceeds uptake by vegetation [8]. This situation thus promotes high nutrient accumulation resulting in acidification and eutrophication of soil and water [9]. The goals of organic farming and livestock production include protecting the environment and pursuing a closed nutrient cycle. However, in free-range farming, nutrient losses are inevitable since ammonia emissions into the air and leaching and runoff of nitrogen, phosphorus and other elements into the soil occur [8]. Total N and P loss is influenced by nutrient uptake by vegetation, the amount and composition of manure deposited, and its concentration and distribution. Key aspects are, therefore, also the number of hens present outside, their dispersal, and the periods when the hens have access to the outdoor pen [10]. In other sectors of free-range livestock production, innovative management strategies have recently been proposed to reduce nutrient losses in the environment, such as temporary stand-off pads [11]. In the poultry sector, to promote better nutrient distribution, hens should use the range as evenly as possible. For this purpose, free-range label organizations recommend structuring the outdoor area with trees and installations that provide shade and protection for the hens. Several studies indicate that structuring elements, roofed shelters, straw bales and trees in the hen run improve the frequency and distribution of birds [12,13,14]. However, there are large individual and flock differences [15]. Therefore, more insight is needed into the motivation of both individual chickens and various strains to use the range and their effect on the health, performance and welfare of the birds.

This study is included in the “FreeBirds—Optimising the use of the free range as the key to improving organic chicken production”. The general aim of the project was to generate more insight into the relationship between chickens’ free-range use and bird health, welfare and performance, as well as soil nutrient load. Furthermore, the project aimed to develop smart tools and management strategies for improving the free-range system in organic poultry production. In more detail, this study focused on the relationship between hens’ free-range use and the manure nutrient load in the soil. This work intends to contribute to farmers’ awareness about the environmental impact of their range management, development and validation of practical solutions and facilities (best practices), stimulating the birds to make optimal use of the range area. This will help farmers to develop more successful husbandry practices and, therefore, obtain better results. To this purpose, monitoring activities were conducted in three Italian organic farms, and the analysis of soil samples at different depths and distances from the henhouse was performed to analyze the dynamics of nutrients in three different cases in Northern Italy, which differ in terms of farm structure, size and pedoclimatic conditions. The evaluation was conducted in three different contexts, in farms with different characteristics, to evaluate three different situations on a full scale. The results will contribute to more sustainable organic poultry production, as well as consolidate consumer acceptance and marketing of organic products, as the knowledge acquired will enable farmers to optimize the birds’ range use according to the intentions of the organic concept.

## 2. Materials and Methods

To determine the effect of the presence of hens on soil quality, monitoring of soil characteristics was conducted for each of the three Italian farms with free-range hens at the end of one of the production cycles, with sampling conducted in the 2020 summer season.

The farms are characterized by the different structures of the run: Farm A with forest and bushes, Farm B with orchards and Farm C with artificial wood shelters. The main soil characteristics of each farm for the 0–30 cm layer are reported in Table 1.

Farm A is a small organic farm of 700 laying hens located at an altitude of 433 m above mean sea level (AMSL) in the mountain area of the province of Reggio Emilia in the Emilia-Romagna region. The observed group consists of 500 hens having access to an outdoor run with an area of 2400 m^2^ (hens’ density: 4.8 m^2^/hen) from the age of 16 weeks. Hens’ age at the time of the farm visit was 60 weeks. Laying hens were housed in a shelter equipped with nests, feeding/drinking facilities and a popup giving the hens access to an outdoor run downhill (i.e., the slope of 20–30%) covered with trees and thick bushes. The outdoor run has been in use for 18 years.

Farm B is a medium size organic farm of 12,000 laying hens located at an altitude of 286 m AMSL in the plain area of the province of Cuneo in the Piedmont region. The observed group consists of 3000 hens having access to an outdoor run with an area of 12,000 m^2^ (hens’ density: 4 m^2^/hen) from the age of 16 weeks. Hens’ age at the moment of the farm visit was 90 weeks. Laying hens were housed in a shelter equipped with nests, feeding/drinking facilities and popups, giving hens access to a flat outdoor run covered with grass and hazelnut trees. The outdoor run has been in use for 20 years.

Farm C is a medium size organic farm of 2600 laying hens, located at an altitude of 850 m AMSL in the mountain area of the province of Bolzano in the Trentino Alto Adige region. The observed group consists of 970 hens having access to an outdoor run with an area of 4000 m^2^ (hens’ density: 4.1 m^2^/hen) from the age of 20 weeks. Hens’ age at the moment of the farm visit was 60 weeks. Laying hens were housed in a shelter equipped with nests, feeding/drinking facilities and popups, giving hens access to a flat outdoor run surrounded by trees and covered with grass. Eight artificial sheds (1 m^2^ large) made of 2 wooden pallets and placed at 4 distances from the hen’s house: 7.5, 15, 30 and 45 m from the nearest popup. The outdoor run has been in use for 15 years.

### 2.1. Soil Sampling and Chemical Analysis

On each farm, the soil was sampled in four different positions, three increasing distances from the hen house (D1—5 m, D2—20 m, D3—50 m) and one area outside the range (*t*-test) not accessed by the animals. For each distance and the external area, soil samples were taken from three different zones (right, middle and left) and two depths, 0–10 cm and 10–30 cm, under the assumption that the upper part of the soil (topsoil) is more affected by external conditions than the soil below. Thus, a total of 3 farms × 4 positions × 3 replicates × 2 depths = 72 samples were collected, 24 for each farm. Soil sampling was performed by using a purpose-built tubular soil sampler, which prevents contamination between layers.

The following chemical parameters were determined for each sample: dry matter, pH, total organic carbon, total Kjeldahl nitrogen, nitrate nitrogen and available phosphorus (Olsen method). For these analyses, the samples were dried in an oven at a temperature between 30 and 40 °C (set temperature 35 °C); then, the samples were sieved at 2 mm (official method of soil chemical analysis, GU 21/10/1999).

The description of the analytical methods is given below:

- pH: 10.0 g of the soil sample dried at 30 °C < T < 40 °C is placed in a 100 mL wide neck Erlenmeyer flask with 25 mL deionized water. This is left to stand for one night after stirring with a glass rod. The reading of the pH value is made by introducing the specific electrode into the suspension and kept in agitation;
- Total organic carbon (TOC): TOC was detected with an internal method, based on the Springer and Klee method. 2 g of the soil sample dried at 30 °C < T < 40 °C is subjected to oxidation with potassium dichromate and concentrated sulfuric acid (20 mL K_2_Cr_2_O_7_ 1 N, 30 mL H_2_SO_4_ + Ag_2_SO_4_ and 5 mL distilled water) under a defined temperature and time conditions (150 ± 3 °C oxidative digestion; duration = 2 h). After that, the sample is cooled and brought to a volume of 250 mL with distilled water. At the same time, it is necessary to prepare a cold blank (using the same amounts of reagents used in digestion, without sampling and heating) for the determination of Mohr salt titer and a hot blank (prepared like the previous ones but subjected to the digestion treatment). TOC is measured by a volumetric method consisting of titration of the potassium dichromate remaining after oxidative digestion with Mohr’s salt-reducing solution. By the difference from the amount of potassium dichromate present in the digested blank, the amount consumed by the organic matter in the sample can be obtained. The titration of the mineralized solution (100 mL taken from the 250 mL flask) is performed, adding distilled water to ensure good immersion of the electrode and stirrer. Determination of the exact titer of the Mohr salt solution is conducted in a similar manner using the cold blank, while the titration of the hot blank is intended to eliminate the error due to the decomposition of the dichromate during the heating of the oxidizing mixture;
- Total Kjeldahl nitrogen (TKN): An aliquot of a sample ground and dried in a ventilated oven at 35 °C ± 5 °C between 2 and 5 g is placed inside Kjeldhal mineralizer tubes and fortified with 10 mL of concentrated H_2_SO_4_ and 5 + 5 mL of H_2_O_2_ in the presence of Kjeldahl method catalyst; mineralization is performed by placing the tube in a Kjeldhal mineralizer at a temperature of 380 °C until the solid residue appears white-grey and the liquid appears colorless. The mineralized residue is then subjected to distillation after basification to release the ammonia produced after digestion, which is captured by a boric acid solution and then titrated with sulfuric acid;
- Nitrate nitrogen: Deionized water is added to a fresh soil sample in variable ratio, then shaken (1 h), centrifugated (10’ at 7000 rpm) and filtrated (0.2 m filter). The sample thus obtained and suitably diluted is introduced into the ion chromatograph with appropriate calibration curves.
- Available phosphorus (Olsen method): 2.5 g of the soil sample dried at 30 °C < T < 40 °C is placed in a 250 mL Erlenmeyer flask with 0.5 g of activated carbon and 50 mL of extracting solution (sodium bicarbonate 0.5 mol/L at pH 8.5). This is shaken for 30’ and filtered. After the addition of 1 mL of sulfuric acid (5 mol/L) and 30 mL of colorimetric reagent to 10 mL of the filtrate in a 50 mL flask, brought to known volume with deionized water, the phosphorus is detected by spectrophotometry by developing the phosphomolybdic complex colored blue.

### 2.2. Statistical Analysis

Chemical data were analyzed with IBM SPSS Statistics v.26 statistical packages. For each farm, sampling depth and parameter, an analysis of variance (one-way ANOVA) and Duncan’s multiple range test (DMRT) as a post hoc test were performed. The differences in concentration between the first (0–10 cm) and the second (10–30 cm) soil layer were assessed for each farm and each parameter using Student’s *t*-tests, excluding the values for the test area not accessed by the animals.

A comparison between the results of the different companies was not conducted since the characteristics of the farms, the size, the context and the geographical location are different.

## 3. Results

### 3.1. Farm A

**Figure 1:**
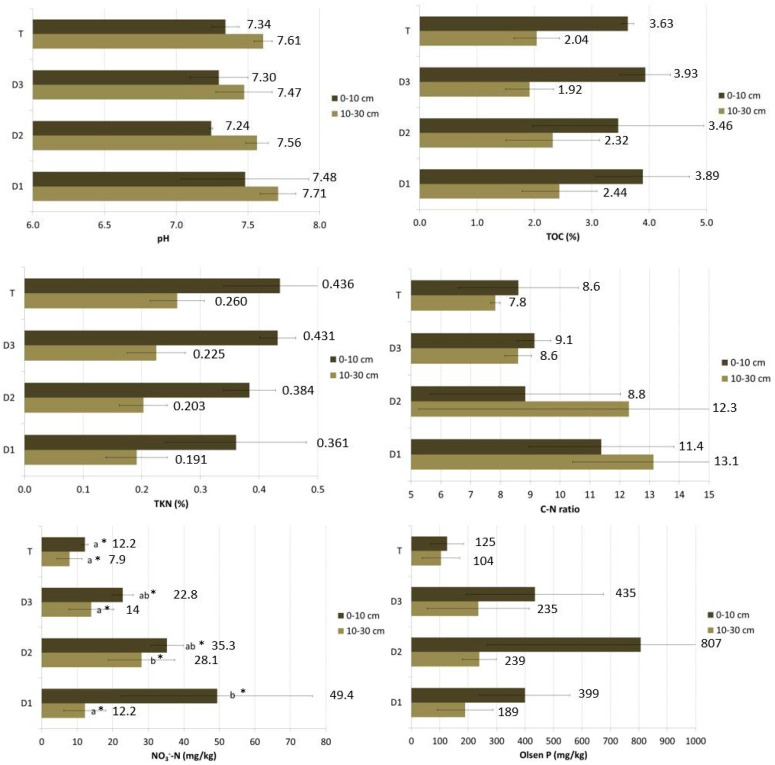
Average values and standard deviation for each sampling position and depth (FA). Different letters correspond to statistically significant differences among the means of measurements of the same depth at different distances. * significant at *p* ≤ 0.05 according to ANOVA-test.

Concerning the parameters analyzed for the two depths, there was a statistically significant difference between the concentrations of the first layer (0–10 cm) and those of the second layer (10–30 cm) for each of the parameters, particularly the differences were greater for total nitrogen and organic carbon (*p* ≤ 0.01), both more concentrated in the surface layer, as well as nitrate nitrogen and Olsen phosphorus, while there was no difference in their ratio (C/N). Figure 1 shows the average values for each sampling position and depth, with the results of the statistical analysis comparing the four positions. The only significant difference between the positions was for nitrate nitrogen.

For the 0–10 cm layer, the difference between D1 and T was statistically significant. NO_3_-N content is 12.2 mg/kg in the T position, while it is 49.4 mg/kg in D1. The maximum value in D1 represents a rather high value for agricultural land. However, data of this order of magnitude are common in heavily fertilized soils. Although there is an increasing trend (D1 content > D2 content > D3 content > T content) among the other distances, there are no statistically significant differences.

In the 10–30 cm layer, the difference was between D2, with the higher value (28.1 mg/kg), and the other three positions.

There was also an increasing trend due to the presence of hens, compared to the test area without animals, for available phosphorus. However, in this case, the differences were not statistically significant, and there were high standard deviations. This parameter reached particularly high values within the range, especially for position D2 (around 800 mg Olsen P/kg). Soil pH is not affected by animal excretions, with small variations among the different sampled points (1–2% of variation).

Somewhat unexpectedly, total nitrogen concentrations seem to have an increasing trend as one moves away from the poultry house, with the highest values in the test area, for both depths. However, the variability of the data is high, and the differences are not significant. Moreover, TOC seems to not be affected by the hens’ presence, and the difference between the D1 value and T value is very small.

### 3.2. Farm B

Concerning Farm B, there was a statistically significant difference between the concentrations of the first layer (0–10 cm) and those of the second layer (10–30 cm) only for total nitrogen (*p* ≤ 0.05) and organic carbon (*p* ≤ 0.01), both more concentrated in the surface layer, while there was no difference in their ratio (C/N).

**Figure 2:**
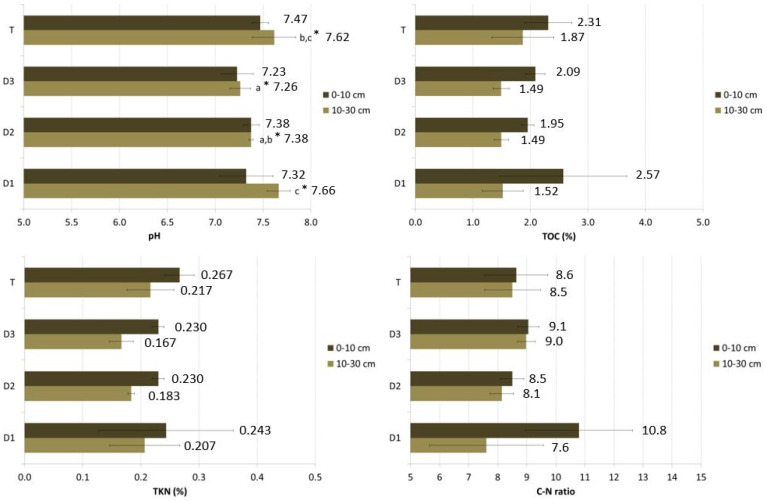

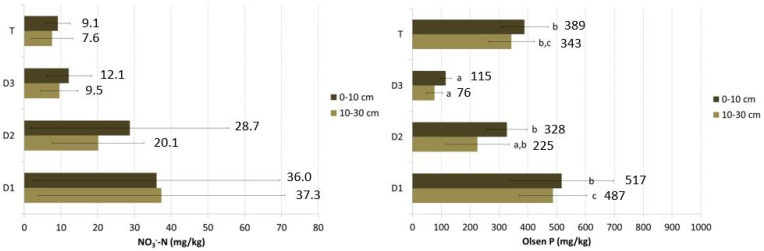
Average values and standard deviation for each sampling position and depth (FB). Different letters correspond to statistically significant differences among the means of measurements of the same depth at different distances. * significant at *p* ≤ 0.05 according to ANOVA-test.

Figure 2 shows the average values for each sampling position and depth, with the results of the statistical analysis comparing the positions.

Significant differences among positions were found only for pH and Olsen phosphorus. Regarding pH, the differences were only for the 10–30 cm layer, while for Olsen phosphorus, the differences were significant for both layers.

Particularly, the pH showed a decreasing trend in values as one moves away from the poultry house (D1 pH > D2 pH > D3 pH); however, T was like D1.

As for Farm A, no statistically significant differences were found between positions for either total nitrogen or organic carbon, parameters which differ only in sampling depth.

The nitrate nitrogen showed a decreasing trend in values as one moved away from the poultry house (D1 content > D2 content > D3 content), but without statistically significant differences due to the high variability of the data. The higher average values were found at the position close to the poultry house (D1, 36 mg/kg for 0–10 cm depth and 37.3 mg/kg for 10–30 cm).

The same trend is found for Olsen P, but in this case, the difference is statistically significant, with clearly decreasing values moving from D1 (517 and 487 mg/kg, respectively, for 0–10) to D3, while a higher value was found in the test area (T), whose high available phosphorus content may be ascribed by past fertilization and/or to flood runoff.

As in the previous case, there are no statistically significant differences in the trend of the TOC and TKN, and the TKN concentration is higher in the test area (Figure 2). TOC at a depth of 10 cm shows its highest level in D1; however, at a depth from 10–30, it is higher in the test area (T).

### 3.3. Farm C

Regarding the analyzed parameters for the two depths, there was a statistically significant difference between the concentrations of the first layer (0–10 cm) and those of the second layer (10–30 cm) only for total nitrogen (*p* ≤ 0.05), which was more concentrated in the surface layer, while there was no significant difference for the other parameters (results not shown). This is probably due to the light texture of the soil, which favors the penetration of elements.

**Figure 3:**
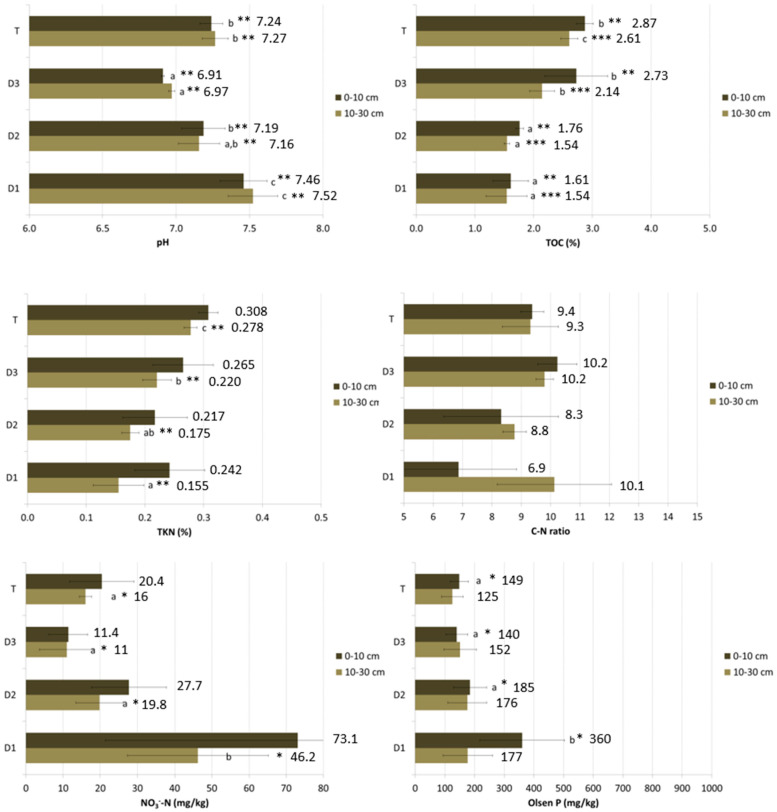
Average values and standard deviation for each sampling position and depth (FC). Different letters correspond to statistically significant differences among the means of measurements of the same depth at different distances. * significant at *p* ≤ 0.05, ** significant at *p* ≤ 0.01, *** significant at *p* ≤ 0.001, according to ANOVA.

Figure 3 shows the average values for each sampling position and depth, with the results of the statistical analysis comparing the positions.

Significant differences among positions were found for pH, total nitrogen, total organic carbon, nitrate nitrogen and Olsen phosphorus. Regarding total and nitrate nitrogen, the differences were only for the 10–30 cm layer, while Olsen phosphorus was only for the 0–10 cm layer. Regarding pH and TOC, the differences were significant for both layers.

Particularly, the pH showed a decreasing trend in values as one moved away from the poultry house (D1 pH > D2 pH > D3 pH), while T was like D2.

The higher values for nitrate nitrogen, reflecting heavily fertilized agricultural soils (around 50 mg NO_3_^−^-N kg), were found at the position close to the poultry house (D1). The same for Olsen phosphorus, with the highest average value (360 mg Olsen P/kg) measured for the 0–10 cm layer at the position close to the poultry house (D1).

As well as in Farm A, somewhat unexpectedly, total nitrogen concentrations seem to have an increasing trend as one moves away from the poultry house, with the highest values in the test area, for both depths. The variability of the data is higher in the layer 0–10 cm than in the underlying 10–30 cm with significant differences. The same is the case for organic carbon, for which the differences are even more pronounced, and the order is confirmed, with an increasing trend as one moves away from the poultry house (D1 < D2 < D3 < T).

## 4. Discussion

The risks of excessive nutrient loading in range area soils appear to be site-specific and probably depend on a combination of different environmental factors [16]. This was also reported in this study, where different trends were found for some parameters (e.g., pH, TKN, TOC and C/N) on the three farms and depending on the sampling depth. In particular, a decrease in pH was observed in all three farms as one moved away from the hen house, with a statistically significant difference in Farm B for the deeper layer and Farm C for both layers. However, at Farm B, at 0–10 cm depth, the pH trend is exactly the opposite.

Total nitrogen seems to have an increasing trend as one moves away from D1, with the highest values in the T-area on each farm. The differences are statistically significant in Farm B and Farm C, both in the deeper layer.

Total organic carbon also has a discontinuous trend, and no clear pattern can be discerned in any farm, except for Farm C, where it increases progressively, moving away from the poultry house, with statistically significant differences in both layers. This is supposed to be due to the hens’ presence, which reduces the vegetation cover of the soil and consequently makes it more prone to organic matter loss; on the other hand, in the test area, the presence of vegetation and root material increases soil organic matter.

Other studies have tried to show the dynamics of these soil parameters; in particular, Bracke et al. [17] report that there are no significant differences in pH and organic carbon between areas where there are likely to be no hens, few hens or many hens, but unlike the present study, soil pH was always < 5.5. In addition, Kratz et al. [8] try to find a correlation between TN, TC and pH with vertical movements of N and P but report that their effect is small or negligible.

On the other hand, nitrate and phosphorus need separate deepening. Concerning these two parameters, a similar trend was found in all three farms; those were consistently higher in sample D1 (5 m from the chicken coop) and decreased when moving away from the hen house. In more detail, the nitrate content in D1 (39 to 73 mg NO_3_^−^-N kg) reflects heavily fertilized agricultural soils. However, on farm A, at a depth of 10–30 cm, the D2 sample has a higher nitrate content than D1. This can be explained by the fact that the first 5 m outside the shed were completely devoid of vegetation and with extremely compacted soil. In addition, at D2 distance, the outside area has a downward slope and the presence of a small wood. These factors certainly influenced both the nutrient dynamics and the increased presence of hens at this distance.

It is well known that in the outdoor run, birds do not use space uniformly and that nutrient supply is concentrated in their preferred areas [8]. Menzi et al. [18] calculated that the fecal nutrient load in the preferred area of a free range is about 11 times higher than the average in the whole free range. In addition, several studies [10,17,19] have reported that hens tend to stay close to the barn resulting in an accumulation of droppings. Therefore, this area is often very nutrient-laden, and this is confirmed by this study. Moreover, this accumulation is even more evident in the most superficial soil layer (0–10 cm). Maffia et al. [20] report that the top layer up to 15 cm deep is considered to be the one most affected by chicken dusting activity. However, in this study, deeper soil layers are also affected by fecal nutrient input, and this is confirmed by Kratz et al. [8], where fecal nutrients by broilers resulted in mineral N accumulation in the soil at a sampling depth of 90 cm. In addition, other previous studies have demonstrated the effect of fecal N load in laying hens [21] and the application of poultry litter on grasslands [22,23] on soil nitrate concentrations up to a depth of 100 cm. In this regard, soil texture and permeability are parameters to be considered. In this study, higher values of nutrient loading were found in the shallower soil samples (0–10 cm), especially on the farm with fine-textured soil (farm A). On farms B and C, the trend is less pronounced, as the sandier soils facilitate leaching and permeability of nutrients, which reach the deeper layers more easily. Therefore, as mentioned above, it is important to study the problem on a case-by-case basis because the results and dynamics may differ and depend on many factors. Several similar studies in the literature report different results with great variability. For example, while Jones et al. [24] found no change in nitrate and phosphate concentrations in groundwater associated with the expansion of an open-air livestock farm in the United Kingdom, Lee et al. [25] concluded that soil-concentrated nutrient zones in poultry farming areas are highly likely to cause environmental problems through leaching and runoff. Similarly, Kratz et al. [8] reported “excessive” concentrations of mineral nitrogen and available phosphorus in the soils of poultry farming areas in Europe and hypothesized environmental risks associated with nutrient volatilization, leaching and runoff. However, it should be considered that all outdoor farms in the present study had trees, shrubs or artificial sheds that may influence the distribution of hens; as reported in many studies [12,14,26], these elements favor hen grazing and a more even distribution of the grassland. For this reason, the differences found in this study between the area proximal to the chicken coop and other more distant sampling areas are smaller overall than in other studies.

Finally, it should be considered that the risks of nutrient loss are related to well-defined site characteristics, such as climate, excess water, soil type, land cover, groundwater depth, precipitation-modifying characteristics, and distance from waterways [27]. Therefore, when site risk factors are low, “nutrient hotspots” (high levels of soil nutrients in small areas) alone do not lead to high nutrient risk [16,27]. It is important to note that the risk of nutrient loss is also highly dependent on the vegetation cover of the soil. Several studies [8,10,14] report that both nutrient loading and the degree of vegetation cover on the land are correlated with the density of laying hen presence and vice versa. In areas with a high density of animals, there is both a high fecal nutrient load and a high degree of vegetation destruction due to hen feeding. Fecal N and P load in parcels can reach or already exceed the nutrient uptake by intact turf even with modest frequency of use (i.e., 25% of the feces produced during the grazing period excreted outside the parcel) by broilers [28]. Therefore, as also reported by Wiedemann et al. [16], it is important to subdivide the various cases according to the degree of risk and adapt appropriate strategies to mitigate the nutrient load on the outdoor cycle and the environmental impact.

## 5. Conclusions

This study shows that the possible environmental impacts deriving from the outdoor run management of an organic laying hen farm are a very delicate and complex issue. Even if the presence of trees, bushes and artificial sheds facilitates the dispersion of birds in the lawn, it remains evident that the area proximal to the house of the hen is characterized by an excessive load of nutrients. However, the environmental risk is site-specific, and it depends on many other factors, both environmental and due to the management (weather conditions, time of day and season, type of soil, hen behavior, size of flock, hen age, etc.). Therefore, future studies should focus on the correlation of several parameters and evaluate the actual nutrient losses due to runoff and leaching.

The high nitrate nitrogen and Olsen phosphorus concentrations, particularly in the area close to the poultry house (5 m) and for the most superficial layer (0–10 cm), represent the most critical point in the results of this study. This high nutrient load leads to a risk in terms of runoff and leaching into surface water (mainly phosphorus) or groundwater (mainly nitrogen). Therefore, actions such as “rotating” hens on different free ranges, establishing temporary spacers, and installing engineered structures covered with bedding materials (e.g., wood shavings, straw or other residues, to be removed and composted at the end of their use) or plant structural elements (trees, bushes, etc.) should be used to reduce the accumulation and risk of nutrient losses, minimizing negative effects on soil and water bodies. Other possible solutions could be phytoremediation or, alternatively, the removal and use of this soil layer as fertilizer for other nitrogen- and phosphorus-poor soils. However, these types of solutions would require a greater effort on the part of the farm, and all the implications would have to be further investigated.

## Figures and Tables

**Table 1:** Main characteristics of the three farm soils at a depth of 0–30cm.

Parameter	Unit	Farm A	Farm B	Farm C
Sand	%	24	46	52
Loam	%	47	40	42
Clay	%	29	14	6
Active CaCO_3_	%	15.9	3.0	1.6
Total CaCO_3_	%	25.0	2.0	2.0
Electrical conductivity	mS/m	101	145	51
Cationic exchange capacity	meq/100 g	22	16	11
Total phosphorus	mg P/kg	782	698	727
Exchangeable potassium	mg K_2_O/kg	640	320	160

## Data Availability

The data presented in this study are available on request from the corresponding author.
